# Validation and Reliability of the Spanish Internet Addiction Test-7 (IAT-7) for Adolescents

**DOI:** 10.3390/ejihpe16020028

**Published:** 2026-02-14

**Authors:** José Antonio Romero-Macarrilla, Robert Bauer, Javier Fernández-Sánchez, Eva Fernández-Sánchez, Iván González-Gutiérrez, José Carmelo Adsuar, Daniel Collado-Mateo

**Affiliations:** 1 Faculty of Teaching Training, University of Extremadura, Avenida de la Universidad, S/N, 10071 Cáceres, Spain; jaromerom01@unex.es; 2 Research Centre in Sports Science (CIDE), Rey Juan Carlos University, 28942 Fuenlabrada, Spain; robert.bauer@urjc.es (R.B.); eva.fernandezsa@urjc.es (E.F.-S.); daniel.collado@urjc.es (D.C.-M.); 3 Faculty of Social and Human Sciences, Universidad Europea del Atlántico, 39011 Santander, Spain; ivan.gutierrez@uneatlantico.es; 4 BioErgon Research Group, Faculty of Sport Sciences, University of Extremadura, 10003 Cáceres, Spain

**Keywords:** IAT-7 scale, problematic internet use, Spanish version, internet addiction, smartphone addiction, psychometric properties

## Abstract

Problematic internet use has been consistently associated with different adverse effects on bio-psycho-social health outcomes. However, there is a lack of consensus in the definition and measures. This study aimed to translate and culturally adapt the Internet Addiction Test–Short Form (IAT-7) into Spanish and to evaluate its validity and reliability among adolescents. A total of 783 participants aged 12–18 years completed the questionnaires while 106 answered again two months later to assess test–retest reliability. Construct validity was examined using confirmatory factor analysis to test the two-factor structure. Convergent and discriminant validity, reliability, and invariance were analyzed. All items showed significant standardized loadings (0.55–0.85; *p* < 0.001), and fit indices supported the two-factor model. Both factors showed adequate convergent validity, while moderate correlation between factors (*ρ* = 0.667) supported discriminant validity. Test–retest reliability was strong (ICC = 0.814), and internal consistency was satisfactory (Cronbach’s *α* = 0.850; McDonald’s *ω* = 0.853). Furthermore, measurement invariance analyses supported the equivalence of the scale across gender and age. In conclusion, the Spanish IAT-7 is a valid and reliable instrument for assessing problematic internet use in adolescents aged 12–18 years.

## 1. Introduction

The rise of digital technologies has profoundly transformed how people in general and adolescents in particular interact, learn, and spend their leisure time (Ibabe et al., 2024). These technologies influence almost every aspect of daily life, including communication and social relationships, educational processes, and entertainment (Benvenuti et al., 2023; Twenge et al., 2019). While the internet offers undeniable opportunities and access to information, excessive or maladaptive engagement has emerged as a growing concern due to the potential negative impact on psychological well-being, academic performance, and social functioning (Afrin et al., 2024; Benvenuti et al., 2023). Although the fifth edition of the *Diagnostic and Statistical Manual of Mental Disorders* (DSM-5; American Psychiatric Association, 2013) only includes Internet Gaming Disorder (IGD) among problematic digital behavior conditions, other terms related to the maladaptive behavior towards the internet, smartphone, social media, videogames, and online gambling have emerged. One of those terms is *internet addiction*, which is conceived as a severe clinical syndrome characterized by compulsivity, tolerance, withdrawal, and continued use of the internet despite negative consequences (Cash et al., 2012). It involves excessive and uncontrolled internet use that negatively influences effective time management, leading to reduced interest and skills in other areas of life (Weinstein & Lejoyeux, 2010).

The prevalence of internet addiction is conditioned by two main factors that may lead to large differences among studies, such as a lack of consensus in the definition and measures, and geographical and cultural differences (Lozano-Blasco et al., 2022). A recent meta-analysis estimated the global pooled prevalence of internet addiction at around 15%, smartphone addiction at 27%, and social media addiction at around 17% (Meng et al., 2022). This prevalence is especially worrying among university students, which is over 40% and even higher among males compared to females (Liu et al., 2025). Furthermore, adolescents may be the most vulnerable population for developing internet addiction due to early access and the social environment. However, the current evidence is largely heterogeneous, showing prevalences between 1.6% to 47.4%, depending on the diagnostic criteria and the geographical and cultural context (Soriano-Molina et al., 2025). These large differences in prevalence emphasize the need for a consensus in the definition of internet addiction and other related terms such as problematic internet use (PIU).

Although some characteristics of substance addictions may be valid for PIU (for instance, internet withdrawal may lead to cortisol secretion and the dopaminergic activity may be comparable to other addictions; Chun et al., 2018; Schimmenti, 2023) in current society, the internet is a core part of many everyday tasks, thus the motives and consequences must be considered (Panova & Carbonell, 2018). In this regard, PIU can cause physical complaints (e.g., eye strain or musculoskeletal issues) and sometimes financial problems (e.g., via online gambling or shopping), but the overall impact is generally less severe than that observed in substance addictions. In the scientific literature, the term *internet addiction* has often been used interchangeably with others such as PIU, but there are relevant differences that should be considered (Hogan et al., 2024). Distinguishing clinically meaningful *addiction* from *problematic use* or *normal usage* is essential for both research and clinical practice. In this regard, the term *internet addiction* remains controversial, as it implies the existence of a clinical disorder that has not been formally recognized by current diagnostic classification systems (American Psychiatric Association, 2013). In this context, a growing body of research advocates for the use of the term PIU, which refers to maladaptive patterns of online engagement that lead to distress or functional impairment. Panova and Carbonell (2018) argue that labeling excessive technology-related behaviors as *addictions* may be conceptually inaccurate, as these behaviors may not involve the level of severity or consequences typically associated with a proper addiction. They propose that *problematic use* better captures the heterogeneity and contextual nature of these behaviors. Following the suggestions by Panova and Carbonell (2018), in the current article, we will use the term PIU instead of internet addiction.

Regarding the consequences of PIU, it has been consistently associated with different adverse effects on bio-psycho-social health outcomes. A recent meta-analysis showed that adolescents with PIU exhibit significantly higher odds of suffering from mental health problems, including suicidal ideation, depressive symptoms, and anxiety (Soriano-Molina et al., 2025; Shiferaw et al., 2025). Sleep disturbances represent another critical consequence, with adolescents experiencing PIU being 1.85 times more likely to report poor sleep quality (Acharya et al., 2025). Furthermore, it is also associated with adolescents’ poorer academic performance (Dou & Shek, 2021), more aggressiveness, and lower psychological well-being and self-esteem (Soriano-Molina et al., 2025). These findings support the multidimensional impact of PIU on adolescents’ overall well-being and highlight the need for comprehensive strategies to assess, prevent, and treat the maladaptive digital behaviors.

To assess this phenomenon, Young (1998) developed the Internet Addiction Test (IAT), which has been widely used, cited more than 200 times every year in Google Scholar between 2013 and 2024. Moon et al. (2018) revealed variability in the dimensionality of the IAT, with studies reporting between one and five factors. Given the methodological limitations of some articles, they suggested that the appropriate factor structure for the IAT consists of either one or two factors. Despite these inconsistencies, Moon et al. (2018) reported good psychometric properties in terms of reliability and validity. However, some problems have been attributed to the IAT. First, a Cronbach’s *α* greater than 0.90 can indicate redundancy within the scale’s items, meaning that some items may assess very similar content, and suggesting that the scale could be shortened without losing measurement quality (Streiner, 2003; Valenti et al., 2025). Furthermore, the reduced time required to answer a shorter version makes it more useful in some situations and evaluations. Considering this, Valenti et al. (2025) validated a reduced version with seven items to reduce redundancy and make it more efficient to use while preserving good psychometric quality. However, this questionnaire has yet to be translated and validated in Spanish. The 12-item version (Pino et al., 2022) is the only reduced version available that is derived from the original IAT.

Therefore, to provide a shorter version of the IAT to be used for Spanish adolescents, the objective of this study was to translate and perform a cross-cultural adaptation of the original IAT-7 into Spanish, as well as to evaluate the validity and reliability of this version among Spanish adolescent high school students. The hypothesis of the current study is that IAT-7 will be a valid and reliable instrument to be used among Spanish adolescents. It is also hypothesized that the IAT internal consistency will be adequate while there will be invariance across gender and age.

## 2. Materials and Methods

### 2.1. Participants

An *a priori* power analysis was conducted for the RMSEA-based test of model fit using the *semPower* (version 2.1.3; Moshagen & Bader, 2024) package in R (version 4.4.2; R Core Team, 2025) using RStudio (version 2025.05.0+496; Posit Team, 2025). For the specified two-factor CFA model (df = 13), assuming a null hypothesis of close fit (RMSEA = 0.05) and an alternative hypothesis of poor fit (RMSEA = 0.08), a minimum sample size of N = 215 was required to achieve a power of 0.80 at *α* = 0.05.

To maximize statistical power and to enhance sensitivity for detecting meaningful differences across multiple fit indices and model parameters, the sample consisted of 783 adolescents aged between 12 and 18 years who completed the questionnaires. A total of 106 adolescents from the whole sample completed the same questionnaire again two months after the initial evaluation to analyze the test–retest reliability of the instrument.

Participants were recruited from educational centers after obtaining prior authorization from the head of the institution. The following inclusion criteria were established: (a) providing informed consent, (b) having the authorization of a parent or legal guardian, (c) being able to read and write in Spanish, (d) having access to an internet-connected device (mobile phone, tablet, or computer) to complete the form, (e) being aged between 12 and 18 years, and (f) being enrolled at a Spanish high school.

The study was approved by the ethics committee of the University of Extremadura. Participants agreed to take part and signed informed consent forms. All procedures were conducted with the consent of the parents or legal guardians of the adolescents. The study followed the updated principles of the Declaration of Helsinki (Resneck, 2025).

### 2.2. Instrument

The short form of the Internet Addiction Test (IAT-7), validated by Valenti et al. (2025), was utilized in the current study. This short version is derived from the original IAT created in 1998 (Young, 1998) and consists of seven items (i.e., items 1, 2, 6, 11, 12, 13, and 16) from the original scale (see Table 1). Participants responded to each item via a 5-point Likert-type scale, ranging from 1 (“Never”) to 5 (“Always”). Higher total scores indicate greater levels of PIU. The scale comprises two factors: (1) Interpersonal, Emotional, and Obsessive Conflict (items 11, 12, and 13), and (2) Online Time Management (items 1, 2, 6, and 16). The validation study was conducted in Italy and involved 463 young adults. In that study, the IAT-7 showed promising psychometric properties with adequate internal consistency and construct validity. They found excellent fit-indices (CFI = 0.994, TLI = 0.983, RMSEA = 0.030, and SRMR = 0.016) and adequate internal consistency (McDonald’s *ω* and Cronbach’s *α* > 0.752; Valenti et al., 2025).

### 2.3. Procedure

To validate the IAT-7 in the Spanish population, a cultural adaptation of the instrument was conducted following the guidelines for cross-cultural adaptation of self-reported instruments proposed by Beaton et al. (2000).

This process included five distinct phases: (1) Initial translation: Two independent translators proficient in both English and Spanish participated in this phase. They also had experience in scientific translation (Harkness et al., 2010). The two translators independently translated the original questionnaire from English to Spanish. (2) Synthesis of translations: Both versions were compared, and differences were discussed to achieve a consolidated single Spanish version. Two experts with scientific knowledge in the field reviewed the final translated version to ensure clarity and preservation of the original meaning (Hambleton et al., 2004). (3) Back-translation: Another translator, proficient in both languages and blinded to the original instrument, translated the consolidated version back into English to check for conceptual consistency (Behr, 2017). (4) Expert committee review: A panel of three experts in the field was formed based on scientific expertise and professional experience with adolescents. They were asked by the principal investigator to review all versions and provide feedback to develop a consensus Spanish version. (5) Field testing: The consensus version was tested with 30 adolescent students to assess clarity and comprehension. Participant feedback indicated that no further modifications were necessary, confirming the adequacy of the Spanish version for subsequent psychometric validation.

### 2.4. Research Procedure

Following approval by the Clinical Research Ethics Committee of the University of Extremadura (Decision No. 171/2025; May 2025), data were collected between June and November 2025 in ten high schools located in different regions of Spain (southern, northern, and western regions). After obtaining authorization from each high-school principal, a member of the research team or a volunteer teacher introduced the study during regular class hours. Data collection was conducted in computer laboratories or regular facilities during 20–30-min sessions. Participants accessed the online questionnaire via QR codes or direct links to a Google Forms survey. All responses were anonymized using randomly generated codes prior to analysis. Parental consent was obtained through information sheets and opt-out forms distributed via the schools’ online education platforms before data collection commenced. The participation was voluntary, and students provided informed assent, with explicit clarification that declining participation would not lead to academic consequences. For the test–retest subsample (*n* = 106), participants completed the survey again approximately two months later (September 2025) using the same link. To enable matching across administrations, these participants temporarily provided identifying information (name and surname), which was removed immediately after responses were linked, and all data were subsequently fully anonymized for statistical analyses.

### 2.5. Data Analyses

The data were analyzed in R (version 4.4.2; R Core Team, 2025) using RStudio (version 2025.05.0+496; Posit Team, 2025), with the *lavaan* (version 0.6-19; Rosseel, 2012; Rosseel et al., 2025), *semTools* (version 0.5-7; Jorgensen et al., 2025), and *semPower* (version 2.1.3; Moshagen & Bader, 2024) packages. Construct validity and reliability were assessed. For construct validity, we conducted a confirmatory factor analysis (CFA) to validate and confirm the two-factor structure of the IAT-7. Given the ordinal nature of the Likert-type scale items, the models were estimated using the robust weighted least squares estimator (WLSMV). To evaluate model fit, we used the robust root mean square error of approximation (RMSEA; <0.08), robust comparative fit index (CFI > 0.90), normed fit index (NFI > 0.90), robust Tucker–Lewis index (TLI > 0.95), and the standardized root mean square residual (SRMR < 0.08) (Hu & Bentler, 1999), which were treated as descriptive guidelines rather than strict decision rules. The chi-square statistics were reported but interpreted cautiously, given the sensitivity to sample size. Given the final sample size (N = 783), the analysis had extremely high sensitivity to detect poor global model fit (RMSEA ≥ 0.08). Accordingly, global fit statistics were interpreted as tests of approximate rather than exact model fit, and model evaluation emphasized the magnitude and pattern of multiple fit indices rather than sole reliance on statistical significance. In addition, a chi-square test was employed to compare the fit of the proposed two-factor model with a one-factor alternative. The difference in chi-square was calculated using a scaled correction (Satorra, 2000), rather than by directly subtracting the chi-square values of the two models.

Convergent validity was evaluated using the standardized factor loadings of each item and their associated z-values, along with the values of composite reliability (CR) and average variance extracted (AVE) for the two factors (Fornell & Larcker, 1981). Discriminant validity was examined using Spearman’s rho (*ρ*) coefficients between the model factors, with validity confirmed when *ρ* < 0.85.

For reliability, internal consistency (McDonald, 2013) and test–retest reliability (Aldridge et al., 2017) were examined. As such, McDonald’s *ω* and Cronbach’s *α* were calculated. In addition, the intraclass correlation coefficient (ICC) of the test–retest was obtained. Absolute reliability was assessed by estimating the standard error of measurement (SEM), computed as SEM = SD × √(1 − ICC), where SD represents the mean standard deviation of the two trials (test and retest). The smallest real difference (SRD) was subsequently determined as 1.96 × SEM × √2. Both SEM and SRD values were also expressed as percentages to facilitate comparisons with previous research.

We examined the measurement invariance of the IAT-7 to test whether the scale was equally appropriate for both gender (adolescent women and men) and age groups. A multi-group confirmatory factor analysis (MG-CFA) was used. Age groups were defined as early adolescence (12–14 years) and middle-to-late adolescence (15–18 years), following previous developmental research. A progressive approach was applied, in which increasingly restrictive models were tested sequentially, including configural, metric, and scalar invariance. Configural invariance assessed whether the same factorial structure was present across groups. Metric invariance tested the equality of factor loadings across groups, indicating whether items contributed similarly to the latent constructs. Scalar invariance tested the equality of item thresholds, allowing for meaningful comparisons of latent means. Model comparisons were evaluated using changes in fit indices, with ΔCFI ≤ 0.01 and ΔRMSEA ≤ 0.015 indicating invariance (Chen, 2007; Putnick & Bornstein, 2016). When full scalar invariance was not supported, partial scalar invariance was examined by freeing a minimal number of item thresholds based on modification indices.

## 3. Results

### 3.1. Sample Characteristics

The sample comprised 397 adolescent women and 386 adolescent men living in Spain and studying in Spanish high schools. The age distribution is presented in Table 2, with a mean age of 14.60 years (SD = 1.65).

### 3.2. Confirmatory Factor Analysis

**Figure 1:**
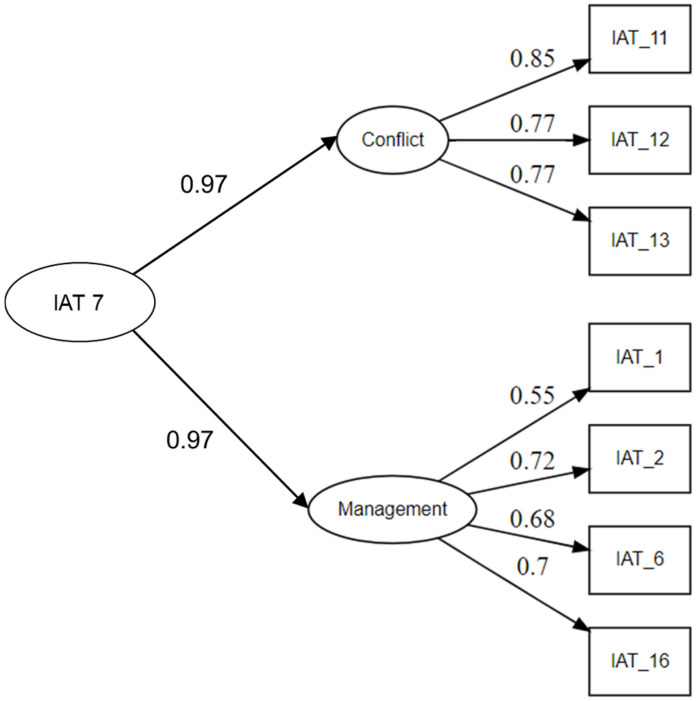
Standardized Factor Loadings for the Two-Factor Confirmatory Model of the Internet Addiction Test-7 (IAT-7).

We conducted a CFA to validate and confirm the two-factor structure of the IAT-7. Figure 1 presents the standardized factor loadings for all items within the two-factor model. All items exhibited standardized factor loadings ranging from 0.55 to 0.85, exceeding the recommended minimum threshold of 0.40 (Kline, 2016), indicating that the observed variables adequately represented the latent constructs (see Figure 1).

Although the chi-square test was significant for the two-factor model (*χ*^2^ = 61.773, *p* < 0.001), this result is expected with large samples and does not necessarily indicate poor fit (Peugh & Feldon, 2020; Stone, 2021). The other fit indices suggested that the two-factor model provided an adequate representation of the data (RMSEA = 0.078, SRMR = 0.030, CFI = 0.974, TLI = 0.958, NFI = 0.986; see Table 3). To further assess the model’s structure, we also tested and compared a one-factor model with the two-factor model. The two-factor model demonstrated a significantly better fit (Δ*χ*^2^ = 12.897; *p* < 0.001), confirming that distinguishing between the two latent factors improved model accuracy, supporting discriminant validity (Anderson & Gerbing, 1988). In addition, the correlation between the two factors (*ρ* = 0.667) indicated a moderate relationship, also supporting adequate discriminant validity.

### 3.3. Convergent and Discriminant Validity

Table 4 presents the convergent validity analyses. All standardized factor loadings (Estimates) were statistically significant (*p* < 0.001), indicating that the items adequately represented their respective latent constructs. Factor 1 (Conflict) showed a CR = 0.799 and an AVE = 0.636, indicating adequate convergent validity (Bagozzi & Yi, 1988). Although the AVE for Factor 2 (Management) was slightly below the recommended 0.50 threshold (AVE = 0.445), composite reliability exceeded 0.70 (CR = 0.734), indicating acceptable convergent validity (Fornell & Larcker, 1981).

### 3.4. Reliability

Test–retest reliability results are presented in Table 5. The ICC values, together with their 95% confidence intervals, are shown for each factor and for the total scale score. The ICC values demonstrated strong stability, with coefficients of 0.814 for Conflict, 0.868 for Management, and 0.876 for the total scale. Furthermore, Cronbach’s *α* values for the retest and McDonald’s *ω* values (representing internal consistency at baseline) are presented. The SEM ranged from 10.45% to 17.10%, and the SRD values varied between 28.98% and 47.39%, suggesting adequate stability between test and retest sessions.

Regarding internal consistency, the analysis revealed overall adequate results for the full scale, with a Cronbach’s *α* = 0.850 and a McDonald’s *ω* = 0.853. Detailed metrics of internal consistency are presented in Table 6, which shows the item-total correlations for each factor and for the full scale, as well as the *α* and *ω* values if each item were removed. Table 6 also shows the descriptive information for each item. All individual items showed statistically significant deviations from normality according to the Kolmogorov–Smirnov test. Given the large sample size, these results might be caused by the high sensitivity of normality tests rather than severe normality violations. Inspection of distributional indices revealed only mild to moderate non-normality at the item level. Skewness ranged from −0.14 (IAT 1) to 0.86 (IAT 13), suggesting slight to moderate positive skewness, indicating a tendency for more concentration of responses at lower scale points with fewer extremely high scores. Kurtosis coefficients were predominantly negative (approximately −0.88 to −0.23), indicating somewhat flatter, platykurtic distributions with lighter tails than the normal distribution. Overall, these findings confirm that responses deviate from strict normality, as expected for Likert scales in large samples, but also that the degree of non-normality is not substantial and is consistent with the use of robust estimation methods (WLSMV) in CFA.

### 3.5. Measurement Invariance Across Gender

The configural model showed good fit (CFI = 0.986, RMSEA = 0.077, SRMR = 0.035), supporting the equivalence of the two-factor structure for adolescent women and men. Metric invariance was also supported, as constraining factor loadings did not substantially worsen model fit (ΔCFI ≤ 0.002; ΔRMSEA ≤ 0.012) (see Table 7).

Full scalar invariance across gender was not supported, as constraining item thresholds resulted in a significant deterioration of model fit compared to the metric model. Inspection of modification indices indicated non-invariant thresholds for a small number of items. After freeing the thresholds of items 16 and 13, partial scalar invariance was achieved. The partial scalar model did not significantly differ from the metric model (Δ*χ*^2^(11) = 9.57, *p* = 0.569), and changes in fit indices were negligible (ΔCFI = −0.001; ΔRMSEA = −0.007), supporting partial scalar invariance across gender (Table 7).

### 3.6. Measurement Invariance Across Age Groups

The configural model demonstrated good fit (CFI = 0.988, RMSEA = 0.073, SRMR = 0.034), indicating a similar factorial structure across age groups (see Table 8). Metric invariance was supported, as constraining factor loadings resulted in negligible changes in model fit (ΔCFI = 0.002; ΔRMSEA = −0.013).

Scalar invariance was also supported across age groups. Constraining both factor loadings and item thresholds did not significantly worsen model fit (Δ*χ*^2^(19) = 26.505, *p* = 0.117), and changes in fit indices remained well below recommended cutoffs (ΔCFI = −0.002; ΔRMSEA = −0.008). These results indicate full scalar invariance across age groups.

## 4. Discussion

In this study, we translated and developed a cross-cultural adaptation of the IAT-7 (Valenti et al., 2025), which is a short version of the well-known 20-item Internet Addiction Test (IAT) developed by Young (1998). As such, we analyzed the validity and test–retest reliability of the translated version with a sample of 783 adolescents enrolled in Spanish high schools. Results showed that the Spanish version of the IAT-7 had adequate psychometric properties, demonstrating good validity and test–retest reliability for assessing problematic internet use (PIU) among Spanish adolescents aged 12 to 18 years.

The comparison between the one-factor and two-factor model indicated that the two-factor structure provided a significantly better fit to the data, confirming that the two latent dimensions—Conflict and Management—are empirically distinct. The moderate correlation between factors further supports adequate discriminant validity, indicating that while they are related, they capture different aspects of the underlying construct measured by the IAT-7. Therefore, the IAT-7 is composed of two factors: (1) Interpersonal, Emotional, and Obsessive Conflict; and (2) Online Time Management. The first factor includes Items 11, 12, and 13 of the original IAT, while the second factor includes Items 1, 2, 6, and 16. All questions address feelings, experiences, and perceptions related to general PIU rather than specific online behaviors. In this regard, it has been suggested that specific potentially PIU, such as online gaming, online gambling, online sexual activity, and online shopping, represent distinct forms of problematic behavior, as the internet provides the medium through which these maladaptive behaviors are expressed, and individuals’ desires and needs are fulfilled (Griffiths, 2018). Therefore, despite the lack of standardization and consensus, the high prevalence of PIU and the negative consequences across multiple dimensions of adolescent and young adult health and well-being have positioned PIU as major public health concern (Mishra et al., 2024).

The two-factor structure identified here offers theoretical insight into the nature of PIU among adolescents. The Interpersonal, Emotional, and Obsessive Conflict factor may reflect emotional dysregulation, preoccupation, and interpersonal strain associated with excessive online engagement, whereas the Online Time Management factor captures difficulties in self-regulation and control over digital behavior. This approach is consistent with the I-PACE model (Brand et al., 2019) and other cognitive–behavioral frameworks for addictive and problematic behaviors (Davis, 2001), which posit that both emotional vulnerability and impaired inhibitory control contribute to addictive tendencies. Thus, the Spanish IAT-7 appears to capture two complementary mechanisms underlying addictive and problematic behaviors in adolescence: affective dependence and deficient self-regulation.

The original IAT questionnaire was developed in 1998 (Young, 1998). Although interactions with the online world have evolved substantially since then, the questionnaire remains widely used, and most items continue to capture relevant perceptions, feelings, and behaviors. However, as pointed out by Valenti et al. (2025), some items required updating. For instance, the item “How often do you check your email before something else that you need to do?” that is included in the original but not in the short form, reflects an outdated behavior pattern. In the pre-smartphone era, checking email required turning on a computer and opening a mailbox, whereas in 2025, most people receive instant notifications on their smartphones, and social communication primarily occurs through social media platforms. Thus, the short form of the IAT not only requires less time to complete but also includes the most relevant and up-to-date items from the original instrument.

From a cross-cultural perspective, the replication of the two-factor structure identified by Valenti et al. (2025) indicates a certain robustness of the IAT-7 framework across diverse populations, suggesting that these dimensions—Conflict and Time Management—may not be culturally bound but rather reflect universal aspects of problematic internet engagement within the scope of this reduced scale. In contrast, full IAT validations have yielded different structures in Italian (two-factors; (Fioravanti & Casale, 2015)) Turkish (four-factors; (Kaya et al., 2016)), and Chinese (three-factors; (Wei et al., 2025)) validations. Consequently, the short version may provide a more parsimonious and culturally adaptable tool for assessing similar aspects across populations. However, future studies may still observe slight variations in item loading due to contextual influences, such as social media use patterns or differences in educational technology integration, warranting further cross-cultural research.

The IAT was first validated in Spanish in 2015 using a sample of college students (Fernández-Villa et al., 2015). Six years later, a 12-item Spanish version was validated with a sample of young adults (mean age = 21.04, SD = 4.72) (Pino et al., 2022). This version also showed a two-factor structure, namely Control and Management of Time and Salience and Neglect of Social Life. It demonstrated high internal consistency and good sensitivity and specificity in differentiating individuals with and without PIU. The present study extends this work by providing a shorter version validated in an adolescent population. Thus, the psychometric properties of the Spanish IAT-7 are specific to adolescents and should not be generalized to young adults. For assessing PIU in individuals older than 18, we recommend using the 12-item version validated by Pino et al. (2022). However, future studies are encouraged to involve a sample with adults older than 18 to validate the IAT-7 for adults.

The developmental relevance of this validation is particularly important. Adolescence represents a critical period for identity exploration, emotional maturation, and peer connection—all increasingly mediated by digital environments (Avci et al., 2024). The Spanish IAT-7 provides a developmentally appropriate tool for capturing adolescents’ PIU. Its brevity and clarity make it especially suitable for school-based screening and prevention programs aimed at identifying early signs of PIU.

An important contribution of this study is the examination of the measurement invariance of the Spanish IAT-7 across gender and age groups. The scale demonstrated configural and metric invariance across both grouping variables, indicating that the underlying factor structure and the meaning of the items are comparable across gender and age groups. Regarding age groups, full scalar invariance was supported, suggesting that adolescents aged 12–14 and 15–18 interpreted and responded to the IAT-7 items similarly. This finding supports the use of the scale for meaningful comparisons of PIU levels across developmental stages. Across gender, full scalar invariance was not achieved; however, partial scalar invariance was supported after freeing the thresholds of a small number of items. Partial scalar invariance is commonly reported in adolescent samples using ordinal response formats and is generally interpreted as reflecting minor differences in response styles rather than substantive differences in the underlying constructs (Chen, 2007; Putnick & Bornstein, 2016). Importantly, the majority of items functioned equivalently across gender, supporting the overall comparability of IAT-7 scores between adolescent women and men. In conclusion, the Spanish IAT-7 is a reliable instrument, as the interpretation of the results is carried out independently of gender and age.

Some limitations must be acknowledged. First, the absence of internet addiction as a diagnostic category in the DSM-5 (American Psychiatric Association, 2013) limits consensus regarding its definition, assessment, prevention, and treatment; thus, in the current study, we used the term PIU instead of internet addiction, following the suggestion by Panova and Carbonell (2018). Second, this study translated and validated the short version of the questionnaire rather than the original 20-item IAT. However, considering the strong validity and reliability of both the original IAT-7 and the Spanish version analyzed here, as well as issues identified in the 20-item version (e.g., item redundancy and outdated content), the selection of the IAT-7 is well justified. Third, the questionnaire was self-administered via computer or smartphone, and no objective measures of online activity were collected. Nevertheless, the IAT assesses psychological and behavioral dimensions rather than the amount of time spent online. Despite these limitations, the study features a rigorous design, including a large and gender-balanced sample. The high internal consistency and test–retest reliability indicate that the IAT-7 can be confidently employed in longitudinal monitoring or intervention evaluation. Its short format enhances feasibility for large-scale surveys, clinical assessments, and national monitoring systems addressing adolescents’ PIU. At a broader level, the adaptation of the IAT-7 supports the development of evidence-based tools that can inform public policy. Reliable data from this instrument may guide national and regional initiatives to promote healthy digital habits, aligning with current public health and educational strategies to mitigate technology-related risks.

## 5. Conclusions

The Spanish version of the IAT-7 is a valid and reliable instrument for assessing PIU in adolescents aged 12–18 years, demonstrating strong psychometric robustness. It comprises the two factors: Interpersonal, Emotional, and Obsessive Conflict and Online Time Management. The psychometric properties of this questionnaire should not be generalized to young adults over 18 years of age; for that population, the 12-item version validated by Pino et al. (2022) is recommended. The use of valid and reliable tools such as the IAT-7 is essential to assess PIU and evaluate the effectiveness of prevention and intervention strategies, given the high prevalence, associated negative outcomes, and expected future increase in this behavior.

## Figures and Tables

**Table 1:** Spanish Version of the IAT-7 and the English Original (Young, 1998; Valenti et al., 2025).

Spanish Version	English Version
1. ¿Con qué frecuencia te das cuenta de que permaneces conectado a internet más tiempo del que tenías previsto inicialmente?	1. How often do you find that you stay online longer than you intended?
2. ¿Con qué frecuencia no prestas la suficiente atención a las tareas domésticas para pasar más tiempo conectado a internet?	2. How often do you neglect household chores to spend more time online?
6. ¿Con qué frecuencia percibes que tus notas o tu trabajo escolar se ven afectados por el tiempo que pasas conectado a internet?	6. How often do your grades or schoolwork suffer because of the amount of time you spend online?
11. Cuando no estás conectado a internet ¿Con qué frecuencia estás pensando en conectarte de nuevo?	11. How often do you find yourself anticipating when you will go online again?
12. ¿Con qué frecuencia temes que la vida sin internet sea aburrida, vacía y sin alegría?	12. How often do you fear that life without the internet would be boring, empty, and joyless?
13. ¿Con qué frecuencia te enfadas, gritas o te muestras molesto/a si alguien te interrumpe mientras estás conectado a internet?	13. How often do you snap, yell, or act annoyed if someone bothers you while you are online?
16. ¿Con qué frecuencia dices «solo unos minutos más» cuando estás conectado a internet?	16. How often do you find yourself saying “just a few more minutes” when online?

*Note.* Each item is rated on a Likert-type scale ranging from 1 = *never* to 5 = *always*.

**Table 2:** Age and Gender Distribution of the Study Participants.

Sociodemographic Variable	*n*	%
Gender		
Women	397	50.70%
Men	386	49.30%
Age		
12	108	13.79%
13	124	15.84%
14	124	15.84%
15	161	20.56%
16	158	20.18%
17	96	12.26%
18	12	1.53%

**Table 3:** Comparison of One-Factor and Two-Factor Models in the Confirmatory Factor Analysis of the IAT-7.

Model	*n*	df	*χ* ^2^	Δ*χ*^2^	RMSEA	SRMR	CFI	TLI	NFI	Interfactor Correlation
Two-factor model	783	13	61.773	-	0.078	0.030	0.974	0.958	0.986	0.667 *
One-factor model	783	14	77.864	12.897 *	0.083	0.034	0.968	0.952	0.982	

*Note.* CFI = Comparative Fit Index; df = degrees of freedom; NFI = Normed Fit Index; RMSEA = Root Mean Square Error of Approximation; SRMR = Standardized Root Mean Square Residual; TLI = Tucker–Lewis Index; * *p* < 0.001.

**Table 4:** Convergent Validity Analysis of the Two-Factor Model of the IAT-7.

Code	*n*	Estimate	*Z*	CR	AVE
Factor 1: Conflict	783			0.799	0.636
IAT 11		0.855			
IAT 12		0.766	37.860 *		
IAT 13		0.767	33.856 *		
Factor 2: Management	783			0.734	0.445
IAT 1		0.551			
IAT 2		0.719	19.696 *		
IAT 6		0.683	19.026 *		
IAT 16		0.702	19.184 *		

*Note.* AVE = Average Variance Extracted; CR = Composite Reliability; * *p* < 0.001.

**Table 5:** Test–Retest Reliability and Internal Consistency Indices of the IAT-7.

Factor	*n*	ICC	95% CI	SEM	SEM%	SRD	SRD%	*α*	*ω*
Conflict	106	0.814	0.727–0.873	1.30	17.10	3.60	47.39	0.814	0.839
Management	106	0.868	0.806–0.910	1.23	10.45	3.40	28.98	0.868	0.760
Total	106	0.876	0.818–0.916	2.09	10.80	5.79	29.95	0.876	0.853

*Note.* ICC = Intraclass Correlation Coefficient; SEM = Standard Error of Measurement; SRD = Smallest Real Difference.

**Table 6:** Item–Total Correlations and Internal Consistency Indices of the IAT-7.

Item	IAT 1	IAT 2	IAT 6	IAT 11	IAT 12	IAT 13	IAT 16
Mean	3.39	2.74	2.54	2.58	2.50	2.12	2.85
SD	0.99	1.06	1.23	1.07	1.25	1.19	1.29
Median	3.0	3.0	2.0	2.0	2.0	2.0	3.0
IQR	1	1	2	1	2	2	2
Skewness	−0.14	0.36	0.36	0.51	0.41	0.86	0.08
Kurtosis	−0.42	−0.45	−0.86	−0.28	−0.88	−0.23	−1.10
Item-Total Correlation (Factors)	0.673	0.732	0.737	0.836	0.853	0.795	0.768
Item-Total Correlation (Full Scale)	0.571	0.696	0.702	0.791	0.734	0.718	0.733
*α* ^a^	0.848	0.828	0.833	0.812	0.824	0.824	0.832
*ω* ^b^	0.852	0.832	0.837	0.814	0.828	0.829	0.836

*Note.* ^a^ *α* value if items were dropped; ^b^ *ω* value if items were dropped.

**Table 7:** Measurement Invariance of the IAT-7 Across Gender.

Model	Δdf	Δ*χ*^2^	*p*	ΔCFI	ΔRMSEA	Conclusion
Configural	-	-	-	-	-	Supported
Metric	5	9.483	0.091	0.002	−0.012	Supported
Scalar	19	48.285	<0.001	−0.007	0.000	Not supported
Partial Scalar	11	9.572	0.569	−0.001	−0.007	Supported

**Table 8:** Measurement Invariance of the IAT-7 Across Age Groups.

Model	Δdf	Δ*χ*^2^	*p*	ΔCFI	ΔRMSEA	Conclusion
Configural	-	-	-	-	-	Supported
Metric	5	8.326	0.139	0.002	−0.013	Supported
Scalar	190	26.505	0.117	−0.002	−0.008	Supported

## Data Availability

The data presented in this study are available upon request from the corresponding author. The dataset is not publicly available due to the privacy of the respondents.

## Abbreviations

The following abbreviations are used in this manuscript:

AVE	Average Variance Extracted
CR	Composite Reliability
CFA	Confirmatory Factor Analysis
DSM-5	Diagnostic and Statistical Manual of Mental Disorders
IAT	Internet Addiction Test
IAT-SF	Internet Addiction Test-Short Form
IGD	Internet Gaming Disorder
ICC	Intraclass Correlation Coefficient
NFI	Normed Fit Index
PIU	Problematic Internet Use
RMSEA	Root Mean Square Error of Approximation
SRD	Smallest Real Difference
SEM	Standard Error of Measurement
SRMR	Standardized Root Mean Square Residual
TLI	Tucker–Lewis Index

## References

[B1-ejihpe-16-00028] Acharya S., Chalise A., Marasine N. R., Paudel S. (2025). Exploring the association between sleep quality, internet addiction, and related factors among adolescents in Dakshinkali Municipality, Nepal. PLoS ONE.

[B2-ejihpe-16-00028] Afrin S., Rahman N. A. S., Tabassum T. T., Abdullah F., Rahman M. I., Simu S. H., Kumar L., Noor K., Vishal F. N. U., Podder V. (2024). The impact of internet addiction on academic performance among medical students in Bangladesh: A cross-sectional study and the potential role of yoga. Annals of Neurosciences.

[B3-ejihpe-16-00028] Aldridge V. K., Dovey T. M., Wade A. (2017). Assessing test-retest reliability of psychological measures. European Psychologist.

[B4-ejihpe-16-00028] American Psychiatric Association (2013). Diagnostic and statistical manual of mental disorders.

[B5-ejihpe-16-00028] Anderson J. C., Gerbing D. W. (1988). Structural equation modeling in practice: A review and recommended two-step approach. Psychological Bulletin.

[B6-ejihpe-16-00028] Avci H., Baams L., Kretschmer T. (2024). A systematic review of social media use and adolescent identity development. Adolescent Research Review.

[B7-ejihpe-16-00028] Bagozzi R. P., Yi Y. (1988). On the evaluation of structural equation models. Journal of the Academy of Marketing Science.

[B8-ejihpe-16-00028] Beaton D. E., Bombardier C., Guillemin F., Ferraz M. B. (2000). Guidelines for the process of cross-cultural adaptation of self-report measures. Spine.

[B9-ejihpe-16-00028] Behr D. (2017). Assessing the use of back translation: The shortcomings of back translation as a quality testing method. International Journal of Social Research Methodology.

[B10-ejihpe-16-00028] Benvenuti M., Wright M., Naslund J., Miers A. C. (2023). How technology use is changing adolescents’ behaviors and their social, physical, and cognitive development. Current Psychology.

[B11-ejihpe-16-00028] Brand M., Wegmann E., Stark R., Müller A., Wölfling K., Robbins T. W., Potenza M. N. (2019). The Interaction of Person-Affect-Cognition-Execution (I-PACE) model for addictive behaviors: Update, generalization to addictive behaviors beyond internet-use disorders, and specification of the process character of addictive behaviors. Neuroscience & Biobehavioral Reviews.

[B12-ejihpe-16-00028] Cash H., Rae C. D., Steel A. H., Winkler A. (2012). Internet addiction: A brief summary of research and practice. Current Psychiatry Reviews.

[B13-ejihpe-16-00028] Chen F. F. (2007). Sensitivity of goodness of fit indexes to lack of measurement invariance. Structural Equation Modeling: A Multidisciplinary Journal.

[B14-ejihpe-16-00028] Chun J. W., Choi J., Cho H., Choi M. R., Ahn K. J., Choi J. S., Kim D. J. (2018). Role of frontostriatal connectivity in adolescents with excessive smartphone use. Frontiers in Psychiatry.

[B15-ejihpe-16-00028] Davis R. A. (2001). A cognitive-behavioral model of pathological Internet use. Computers in Human Behavior.

[B16-ejihpe-16-00028] Dou D., Shek D. T. L. (2021). Predictive effect of internet addiction and academic values on satisfaction with academic performance among high school students in mainland China. Frontiers in Psychology.

[B17-ejihpe-16-00028] Fernández-Villa T., Molina A. J., García-Martín M., Llorca J., Delgado-Rodríguez M., Martín V. (2015). Validation and psychometric analysis of the internet addiction test in Spanish among college students. BMC Public Health.

[B18-ejihpe-16-00028] Fioravanti G., Casale S. (2015). Evaluation of the psychometric properties of the Italian internet addiction test. Cyberpsychology, Behavior, and Social Networking.

[B19-ejihpe-16-00028] Fornell C., Larcker D. F. (1981). Evaluating structural equation models with unobservable variables and measurement error. Journal of Marketing Research.

[B20-ejihpe-16-00028] Griffiths M. D. (2018). Conceptual issues concerning internet addiction and internet gaming disorder: Further critique on ryding and kaye (2017). International Journal of Mental Health and Addiction.

[B21-ejihpe-16-00028] Hambleton R. K., Merenda P. F., Spielberger C. D. (2004). Adapting educational and psychological tests for cross-cultural assessment.

[B22-ejihpe-16-00028] Harkness J. A., Villar A., Edwards B. (2010). Translation, adaptation, and design. Survey methods in multicultural, multinational, and multiregional contexts.

[B23-ejihpe-16-00028] Hogan J. N., Heyman R. E., Smith Slep A. M. (2024). A meta-review of screening and treatment of electronic “addictions”. Clinical Psychology Review.

[B24-ejihpe-16-00028] Hu L. T., Bentler P. M. (1999). Cutoff criteria for fit indexes in covariance structure analysis: Conventional criteria versus new alternatives. Structural Equation Modeling: A Multidisciplinary Journal.

[B25-ejihpe-16-00028] Ibabe I., Albertos A., Lopez-del Burgo C. (2024). Leisure time activities in adolescents predict problematic technology use. European Child and Adolescent Psychiatry.

[B26-ejihpe-16-00028] Jorgensen T. D., Pornprasertmanit S., Schoemann A. M., Rosseel Y. (2025). semTools: Useful tools for structural equation modeling *(Version 0.5-7) [R package]*.

[B27-ejihpe-16-00028] Kaya F., Delen E., Young K. S. (2016). Psychometric properties of the Internet addiction Test in Turkish. Journal of Behavioral Addictions.

[B28-ejihpe-16-00028] Kline R. B. (2016). Principles and practice of structural equation modeling.

[B29-ejihpe-16-00028] Liu X., Gui Z., Chen Z. M., Feng Y., Wu X. D., Su Z., Cheung T., Ungvari G. S., Liu X. C., Yan Y. R., Ng C. H., Xiang Y. T. (2025). Global prevalence of internet addiction among university students: A systematic review and meta-analysis. Current Opinion in Psychiatry.

[B30-ejihpe-16-00028] Lozano-Blasco R., Robres A. Q., Sánchez A. S. (2022). Internet addiction in young adults: A meta-analysis and systematic review. Computers in Human Behavior.

[B31-ejihpe-16-00028] McDonald R. P. (2013). Test theory: A unified treatment.

[B32-ejihpe-16-00028] Meng S. Q., Cheng J. L., Li Y. Y., Yang X. Q., Zheng J. W., Chang X. W., Shi Y., Chen Y., Lu L., Sun Y., Bao Y. P., Shi J. (2022). Global prevalence of digital addiction in general population: A systematic review and meta-analysis. Clinical Psychology Review.

[B33-ejihpe-16-00028] Mishra J., Behera M. R., Mitra R., Samanta P., Mahapatra P. K., Kar S. (2024). Prevalence and impact of internet addiction disorder among adolescents and young adults. The Open Public Health Journal.

[B34-ejihpe-16-00028] Moon S. J., Hwang J. S., Kim J. Y., Shin A. L., Bae S. M., Kim J. W. (2018). Psychometric properties of the internet addiction test: A systematic review and meta-analysis. Cyberpsychology, Behavior, and Social Networking.

[B35-ejihpe-16-00028] Moshagen M., Bader M. (2024). semPower: General power analysis for structural equation models. Behavior Research Methods.

[B36-ejihpe-16-00028] Panova T., Carbonell X. (2018). Is smartphone addiction really an addiction?. Journal of Behavioral Addictions.

[B37-ejihpe-16-00028] Peugh J., Feldon D. F. (2020). “How well does your structural equation model fit your data?”: Is Marcoulides and Yuan’s equivalence test the answer?. CBE—Life Sciences Education.

[B38-ejihpe-16-00028] Pino M. J., Herruzo J., Raya A., Ruiz-Olivares R., Herruzo C. (2022). Development of IAT-12, a reduced Spanish version of the Internet addiction test. Current Psychology.

[B39-ejihpe-16-00028] Posit Team (2025). RStudio: Integrated development environment for R. posit software.

[B40-ejihpe-16-00028] Putnick D. L., Bornstein M. H. (2016). Measurement invariance conventions and reporting: The state of the art and future directions for psychological research. Developmental Review.

[B41-ejihpe-16-00028] R Core Team (2025). R A language and environment for statistical computing.

[B42-ejihpe-16-00028] Resneck J. S. (2025). Revisions to the declaration of Helsinki on its 60th anniversary: A modernized set of ethical principles to promote and ensure respect for participants in a rapidly innovating medical research ecosystem. JAMA.

[B43-ejihpe-16-00028] Rosseel Y. (2012). lavaan: An R package for structural equation modeling. Journal of Statistical Software.

[B44-ejihpe-16-00028] Rosseel Y., Jorgensen T., De Wilde L. (2025). lavaan: Latent variable analysis *(Version 0.6-20) [R package]*.

[B45-ejihpe-16-00028] Satorra A. (2000). A scaled difference chi-square test statistic for moment structure analysis. Psychometrika.

[B46-ejihpe-16-00028] Schimmenti A. (2023). Beyond addiction: Rethinking problematic internet use from a motivational framework. Clinical Neuropsychiatry.

[B47-ejihpe-16-00028] Shiferaw B. D., Tang J., Wang Y., Wang Y., Wang Y., Mackay L. E., Luo Y., Yan N., Shen X., Zhou T., Zhu Y., Cai J., Wang Q., Yan W., Gao X., Pan H., Wang W. (2025). Impact of digital addiction on youth health: A systematic review and meta-analysis. Journal of Behavioral Addictions.

[B48-ejihpe-16-00028] Soriano-Molina E., Limiñana-Gras R. M., Patró-Hernández R. M., Rubio-Aparicio M. (2025). The association between internet addiction and adolescents’ mental health: A meta-analytic review. Behavioral Sciences.

[B49-ejihpe-16-00028] Stone B. M. (2021). The ethical use of fit indices in structural equation modeling: Recommendations for psychologists. Frontiers in Psychology.

[B50-ejihpe-16-00028] Streiner D. L. (2003). Starting at the beginning: An introduction to coefficient alpha and internal consistency. Journal of Personality Assessment.

[B51-ejihpe-16-00028] Twenge J. M., Martin G. N., Spitzberg B. H. (2019). Trends in U.S. Adolescents’ media use, 1976–2016: The rise of digital media, the decline of TV, and the (Near) demise of print. Psychology of Popular Media Culture.

[B52-ejihpe-16-00028] Valenti G. D., Craparo G., Faraci P. (2025). The development of a short version of the internet addiction Test: The IAT-7. International Journal of Mental Health and Addiction.

[B53-ejihpe-16-00028] Wei Z., Hassan N. C., Hassan S. A., Ismail N., Gu X., Dong J. (2025). Psychometric validation of Young’s internet addiction test among Chinese undergraduate students. PLoS ONE.

[B54-ejihpe-16-00028] Weinstein A., Lejoyeux M. (2010). Internet addiction or excessive internet use. The American Journal of Drug and Alcohol Abuse.

[B55-ejihpe-16-00028] Young K. S. (1998). Caught in the net: How to recognize the signs of Internet addiction—And a winning strategy for recovery.

